# Clinical Manifestations and Management of Fibrotic Pulmonary Sarcoidosis

**DOI:** 10.3390/jcm13010241

**Published:** 2023-12-31

**Authors:** Jin Sun Kim, Rohit Gupta

**Affiliations:** 1 Department of Thoracic Medicine and Surgery, Temple University Hospital, Philadelphia, PA 19140, USA; 2 Department of Thoracic Medicine and Surgery, Lewis Katz School of Medicine, Temple University Hospital, Philadelphia, PA 19140, USA; rohit.gupta@tuhs.temple.edu

**Keywords:** pulmonary fibrosis, advanced pulmonary sarcoidosis, sarcoidosis, fibrotic pulmonary sarcoidosis

## Abstract

Fibrotic pulmonary sarcoidosis represents a distinct and relatively uncommon manifestation within the spectrum of sarcoidosis and has substantial morbidity and mortality. Due to the scarcity of research focused on this specific disease subtype, our current understanding of pathogenesis and optimal management remains constrained. This knowledge gap underscores the need for further investigation into areas such as targeted therapies, lung transplantation, and quality of life of patients with fibrotic pulmonary sarcoidosis. The primary aim of this review is to discuss recent developments within the realm of fibrotic pulmonary sarcoidosis to foster a more comprehensive understanding of the underlying mechanisms, prognosis, and potential treatment modalities.

## 1. Background

Sarcoidosis is a complex multisystem inflammatory disease characterized by the formation of noncaseating granulomas that predominantly affects the respiratory system [1,2,3]. While over 60% of sarcoidosis patients have resolution of disease in 2–5 years, the remaining experience chronic disease, including fibrotic change [4]. Sarcoidosis generally exhibits notable demographic disparities, with the highest incidence and prevalence seen in Black patients, particularly among females [5]. A population-based study in the United States found that African Americans with sarcoidosis had a 20% higher rate of pulmonary fibrosis, and African-American women with sarcoidosis had a higher mortality rate at a younger age when contrasted with their Caucasian counterparts [6].

The Scadding staging system is used to assess radiographic stages of pulmonary sarcoidosis, with stage 4 denoting advanced fibrotic changes [7]. Approximately 5.4–19.9% of patients may present with fibrotic disease initially [7,8]. Patients with chronic disease experience increased breathlessness and decreased quality of life as radiographic disease worsens [9]. Advanced pulmonary sarcoidosis (APS) is used to denote the forms of sarcoidosis that cause significant risk of loss of lung function, respiratory failure, or death, and include advanced fibrosis and associated complications as well as pulmonary hypertension [10,11]. Although mortality in sarcoidosis is reported to be less than 5%, mortality in APS ranges from 11–21% [4,11,12,13,14,15]. Most of the poor outcomes attributed to APS are due to fibrotic pulmonary sarcoidosis, an entity that needs to be understood better. In this article, we aim to review recent advances in pathogenesis, clinical presentation, evaluation, and management of fibrotic pulmonary sarcoidosis.

## 2. Pathobiology

### 2.1. Basic Pathophysiology: An Interplay between Genetic and Environmental Factors

**Figure 1:**
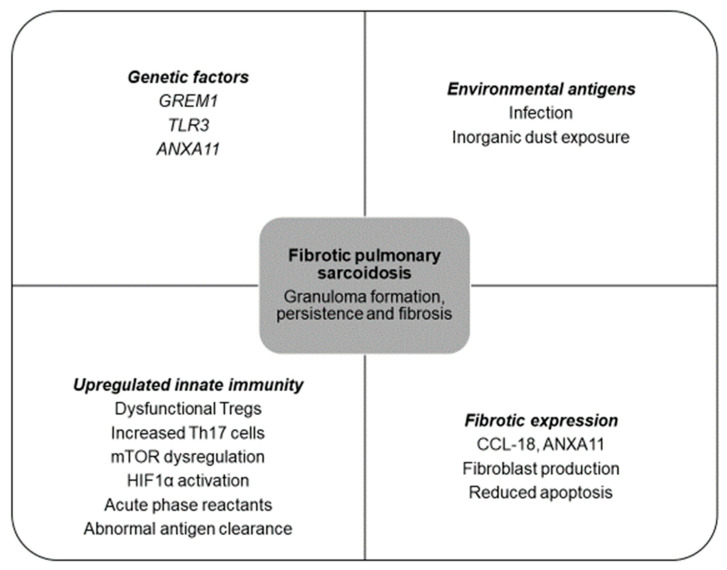
Drivers that may be involved in fibrotic pulmonary sarcoidosis. *GREM1*, gremlin 1; *TLR3*, toll-like receptor 3; *ANXA11*, annexin 11; Tregs, T-regulatory cells; Th17, T-helper 17 cells; mTOR, mammalian target of rapamycin complex 1; HIF1α, hypoxia inducible factor 1; CCL-18, C-C motif chemokine ligand 18.

The pathobiology underlying sarcoidosis and its development into fibrotic disease remains a subject of ongoing research. Sarcoidosis is largely believed to result from a culmination of abnormal immunologic responses following antigen exposure (Figure 1) in a genetically predisposed host. Air pollutants, infectious agents such as mycobacteria and *Cutibacterium acnes*, and exposure to inorganic dust such as silica have been implicated in pulmonary sarcoidosis [16,17,18,19]. Genetic factors have been implicated in the susceptibility and manifestation of the disease, such as HLA-DRB1 on chromosome 6, 5q11.2, 1p22, 3p21-14, 11p15, and 17q21 [5,20,21,22]. Specific alleles of HLA-DRB1 on chromosome 6 may have race-specific associations with varying phenotypes and confer protective effects against disease, while others may be associated with increased disease severity [23,24].

### 2.2. Evolving Knowledge of Pathophysiology in Fibrotic Pulmonary Sarcoidosis

Several genes have been linked to the pathogenesis of fibrotic disease. *GREM1* on chromosome 15q13-q15 encodes a glycoprotein, gremlin, that inhibits bone morphogenic proteins (BMPs) from the TGF-B family [25]. TGF-B, a cytokine secreted by macrophages, T-lymphocytes, and bronchial epithelial cells, promotes extracellular matrix accumulation and inhibits matrix degradation [26]. A study examining *GREM1* variations among sarcoidosis patients with and without fibrosis on chest radiography revealed that carriers of the *GREM1* CC genotype exhibited elevated gremlin levels and were at a 6.4-fold higher risk of developing fibrosis [27]. Genetic variations of TGF-B3 are notably greater in fibrotic patients, and may be associated with the development of pulmonary fibrosis in sarcoidosis [28]. 

Fibrotic pulmonary sarcoidosis has also been linked to specific variants, such as caspase recruitment domain 15 (CARD15) 2104T (702W), CARD15 1761G (587R), and C-C chemokine receptor 5 (CCR-5) [29]. Additionally, a promoter variation in prostaglandin-endoperoxide synthase 2 (PTGS2), −765G>C, has been identified as another potential risk factor for fibrotic disease in sarcoidosis. PTGS2 serves as a regulatory enzyme responsible for synthesizing prostaglandin E2, which is known for its antifibrotic properties. Carriers of the −765C allele were found to exhibit increased susceptibility to sarcoidosis, poorer prognosis, and an increased predisposition to fibrotic disease [30]. 

The pathogenesis of fibrotic pulmonary sarcoidosis remains unclear, but current investigations have identified several potential mechanisms that could elucidate both the fibrotic and inflammatory reactions observed. Pulmonary sarcoidosis is a granulomatous disease characterized by accumulation of lymphocytes and macrophages, inducing granuloma formation. An unknown antigen is first presented to CD4+ T-lymphocytes that trigger T-helper 17 (Th17)-related cytokines, interleukin-17A (IL-17A), regulatory T-cells, and tumor necrosis factor (TNF), a proinflammatory cytokine, to produce granulomas [31]. Granulomas may spontaneously resolve or persist, and may progress to fibrosis via high levels of TNF and mononuclear phagocytes (MNPs) and activation of fibroblasts, myofibroblasts, and collagen formation [32]. 

Chronic fibrosis is thought to be the culmination of increased Th17 cells and primed monocyte-derived macrophages (toll-like receptor-3 (TLR3) polymorphism, type 1 interferon signaling) responding disproportionately to an insult [33]. In particular, monomorphisms in *TLR3* have been implicated in fibrotic pulmonary sarcoidosis, resulting in reduced TLR3 function in innate immune responses and reduced apoptosis of fibroblasts [33,34]. This response drives production of chemokine ligand 18 (CCL-18), which induces fibrogenesis [33]. CCL-18 is associated with fibrotic pathogenesis in IPF, with increased mortality and fibrotic burden on imaging [35,36]. Another protein, annexin A11 (ANXA11), is a calcium-dependent protein involved in innate immunity and cell apoptosis. A small study found a correlation between a minor allele in the *ANXA11* gene and African Americans with fibrotic pulmonary sarcoidosis, and suggested *ANXA11* polymorphism may lead to persistence of Th1 and Th17 cells, resistance to apoptosis, and persistence of granuloma [24,33].

Increased production of CCL-18 from macrophages attracts activated CD4 T-cells and increases transforming growth factor-beta (TGF-β) secretion, enhancing Th17-mediated inflammation. Th17 expresses IL-17A, a proinflammatory cytokine that drives fibrosis and causes corticosteroid resistance [37]. One study found higher bronchoalveolar lavage (BAL) IL-17 levels in patients with pulmonary sarcoidosis without disease resolution, but this was not studied in patients with or without fibrotic disease [38]. Another acute phase reactant is serum amyloid antigen (SAA), which has been shown to induce Th17 response, chronic inflammation, and fibrosis [39]. It can stimulate the production of Th1-mediated granulomatous inflammation via TNG, IL-10, and IL-18, and has been shown to correlate positively with fibrotic disease in chronic fibrotic sarcoidosis [40,41].

Regulatory T-cells (Tregs) are a specialized subset of CD4+ T-cells involved in immunosuppression via production of inhibitory cytokines such as interleukin-10 (IL-10), inhibitory receptors such as cytotoxic T-lymphocyte-associated protein 4 (CTLA-4), and deplete interleukin-2 (IL-2) [42]. Prior studies have found lower numbers of Tregs in BAL and Treg dysfunction in patients with active sarcoidosis [43,44,45]. In both active and fibrotic sarcoidosis patients compared with IPF patients, a recent study found an imbalance of Tregs and Th17.1 cells in peripheral blood and BAL fluid, with lower frequency of Tregs but high Th17.1 in BAL and higher frequency of Tregs but low Th17.1 in peripheral blood [46]. The authors suggest that an increased proportion of circulating Tregs was associated with fibrotic disease on radiography, and the lung microenvironment may affect immunological pathogenesis of sarcoidosis [46].

Another pathway that may contribute to granuloma formation and Th17 differentiation is a dysregulation of the mammalian target of rapamycin (mTOR) pathway. mTOR regulates autophagy and growth in response to stressors [47]. Defects in mTOR-related pathways may inactivate autophagy, decrease pathogen clearance, and cause granulomatous formation and persistence [48]. In fibrotic pulmonary sarcoidosis, mTOR complex 1 (mTORC1) remains upregulated, impairing antigen clearance and promoting excess granulomatous formation [33,49,50]. 

Recently, the hypoxia-induced factor 1-alpha (HIF1α) pathway has garnered attention. A recent investigation found that when exposed to hypoxic conditions, monocyte-derived macrophages increase their proinflammatory response and reduce antigen presentation, leading to a reduction in T-cell response [51]. Through the secretion of profibrotic factor plasminogen activator inhibitor-1 (PAI-1), this process may promote development and persistence of granulomas in active sarcoidosis, reduce fibrinolytic activity, and ultimately contribute to the development of fibrotic disease [51]. 

## 3. Clinical Manifestations

Prior studies have found that the average age of presentation with fibrotic pulmonary sarcoidosis is in the fourth decade of life [7,12]. Up to 20% of patients can present with fibrotic disease at initial presentation, but chronic disease may develop in 20–25% of patients with a prior diagnosis of sarcoidosis [7,52,53]. Clinical symptoms are nonspecific and include dyspnea (80%), cough (51.4%), hemoptysis (2.8%), sputum production (18.3%), crackles (28.2%), digital clubbing (6.3%), and wheezing (5.6%) [12].

### 3.1. Imaging

**Figure 2:**
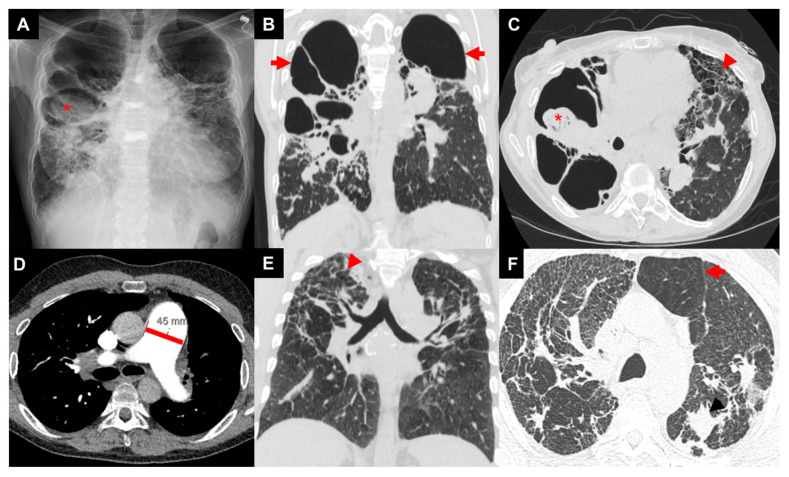
Images of three patients with advanced pulmonary sarcoidosis: patient 1 with biapical cavities and mycetoma (asterisk) on chest X-ray (**A**), large biapical bronchiectatic cavities (arrows) on coronal image of CT chest (**B**), right apical mycetoma (asterisk) and extensive left-sided upper-zone predominant fibrosis (arrow head) of anterior lung on axial image of CT chest (**C**); patient 2 with enlarged pulmonary artery diameter (45 mm) on axial image of CT chest (**D**); patient 3 with bilateral irregular reticular and nodular fibrosis (arrow head) on coronal image of CT chest (**E**) with air trapping (arrow) on axial image of CT chest (**F**).

On chest radiography (CXR), Scadding stage 4 is defined by the presence of pulmonary fibrosis, as mentioned above (Figure 2) [7]. Patients may have upper-lobe-predominant linear opacities projecting from the hilum with dilated airways [54]. High-resolution computed tomography (HRCT) gives a more comprehensive understanding of anatomic changes. Three major patterns of fibrotic sarcoidosis can be identified: central bronchial distortion, peripheral upper zone honeycombing, and diffuse hilar linear opacities (Figure 2) [11,55,56]. Fibrocystic opacities may track along the airways from the hilum to peribronchovascular and fissural regions [11]. HRCT may show subpleural honeycombing, fibrocystic lesions larger than traditional honeycombing, paracicatricial emphysema, and development of mycetomas [11,57]. Granulomatous infiltration of the airways will cause airway distortion, airway angulation, and diffuse wall thickening [7,56]. HRCT can also help screen for sarcoidosis-associated pulmonary hypertension by using a ratio of main pulmonary artery diameter/ascending aorta diameter (MPAD/AAD) greater than 1, evaluating for a dilated pulmonary artery greater than 30 mm (Figure 2), and using a ratio of the diameter of the main pulmonary artery/body surface area (MPA/BSA) greater than 16 [3,11,58]. 

**Figure 3:**
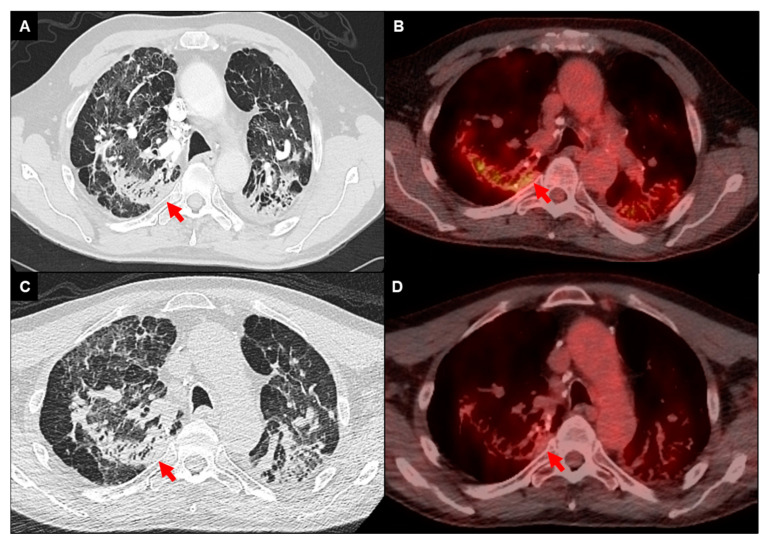
FDG-PET/CT imaging of a 68-year-old male with fibrotic pulmonary sarcoidosis on chronic methotrexate with metabolically active infiltrates (arrows) in the right upper lobe and left upper lobe extending into the pleura (**A**,**B**); reduced density and activity on follow-up imaging 12 months after the addition of anti-TNF-alpha inhibitor (**C**,**D**).

Fluorodeoxyglucose positron emission tomography integrated with computed tomography (FDG-PET/CT) in combination with cardiac MRI is predominantly used for the diagnosis and management of cardiac sarcoidosis [3,59]. In pulmonary sarcoidosis, FDG-PET/CT has exhibited a high sensitivity rate ranging between 94% and 100% in identification of ongoing inflammatory processes (Figure 3) [60,61]. Few studies have investigated its diagnostic use in pulmonary sarcoidosis [62,63,64]. One retrospective study involving 95 patients, with 85% demonstrating signs of fibrotic disease, found that the severity of pulmonary involvement as assessed by HRCT and lung function parameters was associated with increased FDG uptake at a threshold standardized uptake value (SUVmax) of greater than or equal to 2.5 [63]. Another study assessed the role of FDG-PET/CT in comparison with HRCT to identify sarcoidosis activity, and found a discordance rate of greater than 50% between FDG uptake and pathologic changes on HRCT. The presence of active nodal disease, active parenchymal changes, and disease recurrence in extrapulmonary regions were additional findings noted on FDG-PET/CT not discernible on HRCT [64]. 

In the context of disease activity monitoring, a limited number of studies found that patients who exhibited reductions in SUVmax values following glucocorticoid therapy experienced lower rates of relapse, in contrast to individuals without reduction in SUVmax, who notably had higher relapse rates (Figure 3) [65,66]. To date, there is no SUVmax threshold that is validated to denote disease activity or recommendations for use of FDG-PET/CT in determining anti-inflammatory treatment for fibrotic pulmonary sarcoidosis. Future studies are needed to determine optimal utility of FDG-PET/CT imaging in fibrotic pulmonary sarcoidosis. 

### 3.2. Pulmonary Function Testing

On pulmonary function testing (PFT), fibrotic sarcoidosis presents with varying degrees of gas-exchange, airflow-obstruction, ventilatory-restriction, and mixed defects [12]. One study found associations between HRCT anomalies and pulmonary function testing, revealing a connection between restrictive defects and reduced diffusion capacity with interstitial fibrosis and subpleural honeycombing, while airflow obstruction correlated with bronchial distortion. Linear opacities without septal changes were found to have the least functional impairment [55]. Patients with fibrotic disease were shown to have a higher prevalence of mixed ventilatory defects, lower diffusion capacity for carbon monoxide, and higher mortality in another study [67]. A recent study characterizing different pulmonary function phenotypes in sarcoidosis found that fibrocystic patterns on chest imaging (*n* = 22) were more commonly seen in Black individuals, and patients with fibrocystic patterns had a greater degree of restriction and mixed pulmonary function phenotypes than patients with nonfibrotic pulmonary sarcoidosis [68]. The findings of this study emphasize that fibrotic disease is linked to a higher prevalence of restrictive and mixed defects [68]. On 6-min walk tests, individuals with fibrotic sarcoidosis may have reduced walk distance, which has been associated with increased mortality, sarcoidosis-associated pulmonary hypertension, reduced forced vital capacity, and exertional hypoxia [69,70]. 

### 3.3. Serum Biomarkers

Inflammatory biomarkers have been proposed as a method to monitor disease activity and treatment response in pulmonary sarcoidosis. These biomarkers have not been studied in the setting of fibrotic disease, and larger prospective studies are needed to assess clinical utility. Nevertheless, research indicates promising results for a few of these biomarkers, such as serum angiotensin-converting enzyme (ACE), human chitotriosidase, C-reactive protein (CRP), and Krebs von den Lungen-6 (KL-6).

Serum ACE is a glycoprotein produced by alveolar macrophages that converts angiotensin I to angiotensin II in the renin-angiotensin pathway and degrades bradykinin. Granulomas express alveolar macrophages, and serum ACE levels may reflect granulomatous burden [71]. ACE levels are currently the most frequently used laboratory testing in sarcoidosis as a marker for disease activity, although they are neither sensitive nor specific [12,72,73,74]. High serum ACE levels may be seen in patients with greater HRCT abnormalities, including ground-glass opacities, interlobular septal thickening, nodularity, and consolidation [75]. They may be used to monitor treatment effects in sarcoidosis patients. An observational cohort study assessing treatment response with methotrexate by measuring serum ACE and soluble IL-2 receptor (sIL-2R), a marker of T-cell activation, found high baseline levels of ACE correlated with lung function improvement after treatment; and decreases in ACE and sIL-2R after treatment correlated with improved lung function, especially with change in DLCO [73]. In addition, T-helper type 1 cells secrete IL-2 and bind to IL-2R, stimulating T-cell proliferation [76]. sIL-2R is a marker of T-cell activation, whereas ACE reflects total body granulomas. In this study, ACE had a greater correlation with lung function change after methotrexate therapy than sIL-2R [73]. CRP is a proinflammatory acute phase reactant elevated in chronic sarcoidosis, and elevated baseline values may correlate with disease severity, physiologic progression of disease, and treatment response [77,78]. CRP may be useful in monitoring disease activity but requires validation. Further research of serum biomarkers is needed on the clinical utility of these in sarcoidosis in general as well as fibrotic pulmonary sarcoidosis.

## 4. Prognosis

The presence of fibrosis on high-resolution computed tomography (HRCT) scans indicates a poorer prognosis, disease progression, and an elevated risk of mortality [13,55,58,79]. One study proposed a clinicoradiological staging system using HRCT patterns and composite physiological indices (CPI, a weighted index of lung function variables) to determine prognosis in pulmonary sarcoidosis [58]. The staging system was composed of CPI, main pulmonary artery diameter to ascending aorta diameter ratio (MPAD/AAD), and fibrosis threshold of ≥20% [58]. The staging system was found to be straightforward yet reliable for identifying patients with increased risk of mortality [58]. The results further emphasized that CPI was the strongest predictor of mortality [58].

A prospective study in fibrotic pulmonary sarcoidosis evaluated the feasibility of employing percent fibrosis on HRCT, reduced DLCO, or increased CPI score to predict a clinal worsening event over an 18-month study period [80]. A clinical worsening event was defined as death, lung transplant, or greater than absolute 10% drop in percent predicted FVC [80]. Though the study was underpowered at 16 participants due to poor enrollment, it found that individuals with at least 20% fibrosis on HRCT and DLCO less than 30% predicted were more likely to experience a clinical worsening event [80]. 

In a recent study, HRCT features of fibrotic pulmonary sarcoidosis and its impact on pulmonary function and survival were assessed [81]. The study found that the presence of over 20% fibrosis and basal subpleural honeycombing were predictive of deteriorating pulmonary function and worse survival in fibrotic pulmonary disease [81]. Moreover, the researchers found that independent predictors of poor survival included basal subpleural honeycombing, DLCO < 40%, and White race [81]. This is the first study to assess patterns of fibrosis with mortality.

Associations between race and survival have been made by prior studies. As mentioned earlier, a United States population-based study found increased rates of pulmonary hypertension and pulmonary fibrosis in African Americans, and a significantly disproportionate increase in mortality amongst young African-American women compared with their Caucasian counterparts [6]. The recent finding of higher mortality in White race as noted above was shown after controlling for extent of fibrosis, fibrotic pattern on HRCT, presence or absence of sarcoidosis-associated pulmonary hypertension, age, and study location. The uncertainty surrounding the relationship between race, sex, and mortality in fibrotic pulmonary sarcoidosis underscores the need for additional research.

In a retrospective study conducted in France, individuals with fibrotic pulmonary sarcoidosis displayed a mortality rate of 11.3% over an average follow-up period of seven years [12]. Respiratory complications accounted for 75% of patient deaths, while 31.2% were attributed to pulmonary hypertension, and 25% were linked to chronic respiratory failure [12]. Other complications as contributory causes of death included extrapulmonary cardiac involvement, immunosuppressive therapy, and aspergilloma infection [12]. On univariate analysis, the authors found New York Heart Association [82] (NYHA) functional class, forced expiratory volume in 1 s (FEV1) below 63% predicted, forced vital capacity (FVC) below 72% predicted, total lung capacity (TLC) below 74% predicted, diffusion capacity of carbon monoxide (DLCO) below 58% predicted, room-air arterial oxygen tension (PaO2) below 81 mmHg, and the presence of pulmonary hypertension exhibited a significant association with increased risk of mortality [12]. 

## 5. Management

**Figure 4:**
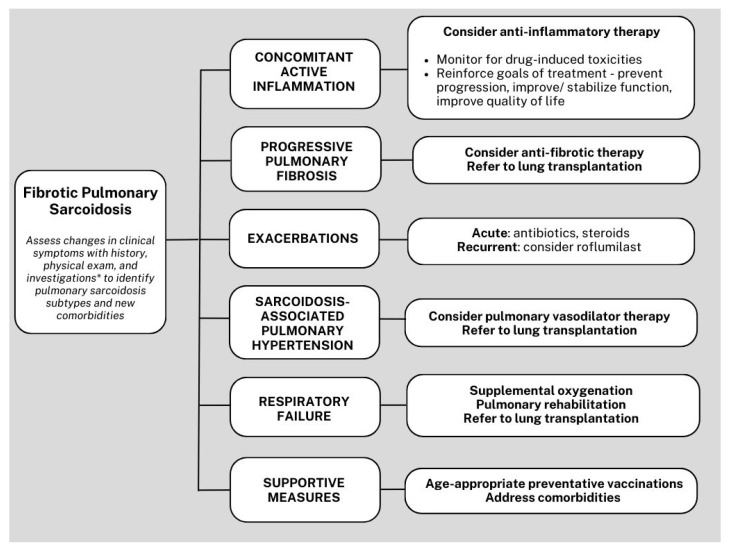
Simplified algorithm for the management of fibrotic pulmonary sarcoidosis. *Investigations with pulmonary function test, 6-min walk test, high-resolution computed tomography, echocardiogram, and fluorodeoxyglucose positron emission tomography integrated with computed tomography.

Management of fibrotic pulmonary sarcoidosis is challenging, largely due to the lack of standardized therapy and variability in presentation and evolution of the disease and needs long-term studies into treatment options. Treatment decisions are often guided by clinical experience and expert opinion. In general, a comprehensive approach involves the integration of various diagnostic tools, including serum biomarkers, PFT, 6MWT, imaging studies, and echocardiography. These assessments can be used to monitor disease progression, identify exacerbations and new complications such as pulmonary hypertension, and progressive respiratory failure. A personalized and multidisciplinary treatment strategy is necessary to address the complexities of the disease, manage comorbidities, deliver supportive care, and consider the possibility of lung transplantation (Figure 4). The next few paragraphs cover the basics of management of patients with fibrotic pulmonary sarcoidosis, but details can be found in corresponding sections of this series.

### 5.1. Anti-Inflammatory Therapy 

Anti-inflammatory agents may preserve or improve lung function, aid in symptom management, and prevent progression of disease in certain patients with fibrotic pulmonary sarcoidosis [12,83]. However, identifying which patients would benefit from treatment remains uncertain. Due to a lack of evidence-based therapies in fibrotic pulmonary sarcoidosis, the Delphi consensus and the ERS clinical practice guidelines on treatment of pulmonary sarcoidosis have not focused on this subset [83,84]. Currently, anti-inflammatory therapy in the setting of fibrotic pulmonary sarcoidosis is done in a case-by-case scenario.

In patients with acute or chronic disease, the primary method for managing inflammation involves the use of glucocorticoids (prednisone 20–40 mg daily) as the initial treatment [83,84]. As clinical symptoms resolve, glucocorticoids are rapidly tapered to doses less than 10 mg or to the lowest effective dose. In cases where glucocorticoids are unable to be tapered, the addition of antimetabolites with methotrexate or azathioprine as second-line agents is considered [83,84,85]. In the context of progressive fibrotic disease, prior use of methotrexate may pose a challenge by raising concerns about pulmonary toxicity, necessitating a thorough evaluation of patients for potential adverse effects. If feasible and without financial barriers, FDG-PET/CT may be useful in identifying areas of inflammation. If unavailable, other disease-modifying agents may have to be chosen. Anti-TNF-alpha agents such as infliximab can also be considered and have been shown to improve or maintain FVC [86,87]. In certain situations with ongoing disease progression, repository corticotrophin injections, rituximab, and JAK inhibitors can be explored, although a consensus is yet to be established [83]. The potential for adverse effects induced by chronic glucocorticoids and chronic immunosuppressive agents requires frequent monitoring and vigilance in identifying drug-induced toxicities. Relapse after discontinuation of therapy can occur, and patients require clinical monitoring. Use of anti-inflammatory agents in the setting of fibrotic pulmonary sarcoidosis needs more evidence. While there are multiple agents being studied in the management of pulmonary sarcoidosis, most of these studies exclude patients with fibrosis >20%. It would be intriguing to see how these agents potentially impact the course of chronic pulmonary sarcoidosis and fibrotic pulmonary sarcoidosis.

### 5.2. Sarcoidosis-Associated Bronchiectasis 

Patients with advanced pulmonary sarcoidosis can develop granulomatous infiltration of the airways, causing fibrotic changes with airway distortion and traction bronchiectasis. Airway abnormalities, along with chronic inflammatory treatments and poor mucociliary clearance, create optimal environments for infections and mycetomas [88,89,90]. 

### 5.3. Acute Pulmonary Exacerbations of Sarcoidosis

There is no consensus on the definition of acute exacerbations in sarcoidosis. Exacerbations have been described in the literature as new or worsening pulmonary symptoms with a decline in FVC or FEV1 for greater than one month, and exclusion of alternative causes [91,92]. Exacerbations may be related to bronchiectasis, infection, and impaired immune response, and require treatment with antibiotics and/or glucocorticoids [88]. In patients with fibrotic sarcoidosis, a small trial (*n* = 38) found that patients with greater than two exacerbations who were treated with roflumilast, a phosphodiesterase-4 inhibitor, had improved FEV1 in subsequent visits and quality of life than those treated with placebo [93]. A larger prospective trial confirming these results would provide valuable insights into the efficacy of roflumilast and could help establish a more standardized approach in identifying and managing acute exacerbations.

### 5.4. Infections

Infections such as aspergillus, mycobacteria, cryptococcus, nocardia, and histoplasma can complicate clinical course and increase morbidity and mortality in patients with sarcoidosis [94,95]. Mycetomas, particularly chronic pulmonary aspergillosis, have been reported in 3–12% of patients with APS [96]. Though frequently asymptomatic, they can cause life-threatening hemoptysis and may require long-term antifungal therapy, bronchial artery embolization, and surgical resection [97]. 

### 5.5. Sarcoidosis-Associated Pulmonary Hypertension

Sarcoidosis-associated pulmonary hypertension (SAPH) is classified into World Health Organization (WHO) group 5 and has been noted to be in 73.8% of patients with advanced pulmonary sarcoidosis awaiting lung transplantation [98]. It is a major cause of morbidity and mortality in APS and a predictor of lung transplant waitlist mortality [13,79,98,99]. Not all pulmonary vasodilator therapies may be appropriate, and treatment decisions regarding pulmonary vasodilator therapy should be made by experts with clinical experience in SAPH due to the multifactorial nature of SAPH and limited double-blind placebo-control trials in patients with precapillary SAPH. 

### 5.6. Antifibrotic Therapy

There are no established guidelines for the use of antifibrotic therapies in the context of fibrotic pulmonary sarcoidosis. Targeted treatments are considered on an individualized basis and continue to be investigated. Insights from the INBUILD trial demonstrated that nintedanib decreased the rate of decline in FVC in progressive pulmonary fibrosis. However, the trial was underpowered for fibrotic sarcoidosis (*n* = 12), and nintedanib needs to be examined in a larger cohort [100]. Similarly, the RELIEF trial showed that pirfenidone had a slower decline in percent predicted FVC, but the study was terminated prematurely due to challenges related to slow recruitment in non-IPF progressive fibrotic lung disease [101]. Moreover, the study excluded patients with sarcoidosis, and the results limit the applicability to this cohort [101]. The PirFS trial, initially designed as a double-blind placebo controlled trial to assess the antifibrotic effect of pirfenidone on fibrotic pulmonary sarcoidosis, was subsequently converted to a phase-4 feasibility trial due to poor enrollment during the COVID pandemic [80]. Preliminary results suggested the potential use of DLCO < 40% predicted as an inclusion criterion for evaluating the efficacy of antifibrotic agents as these patients reached the defined time to clinical worsening [80]. Due to the inflammatory basis of fibrosis in pulmonary sarcoidosis and potential improvement in symptomology and physiologic parameters, the exact role of antifibrotic therapy in fibrotic pulmonary sarcoidosis is unclear at this time. Currently, there is no consensus on whether antifibrotic therapy should be used alone, in conjunction with, or after anti-inflammatory therapy has failed to slow the progression of fibrosis in patients with fibrotic pulmonary sarcoidosis. This needs to be decided on an individual basis. 

### 5.7. Supportive Management

Supportive management, including pulmonary rehabilitation, preventative vaccinations, and supplemental oxygen therapy, may improve overall wellbeing for individuals affected by fibrotic pulmonary sarcoidosis. Pulmonary rehabilitation is a comprehensive program tailored to each patient’s needs, and involves personalized evaluations, exercise training, educational sessions, and behavioral modifications aimed at enhancing overall wellbeing [102]. Studies primarily conducted among patients diagnosed with chronic obstructive pulmonary disease (COPD) have demonstrated significant improvements in mortality, exercise capacity, overall quality of life, and efficient utilization of healthcare resources [103,104,105,106]. Emerging evidence suggests that individuals with interstitial lung disease, pulmonary hypertension, and those undergoing evaluation for lung transplantation derive benefit as well in exercise tolerance and decreased dyspnea [107,108,109]. According to the latest guidelines from the European Respiratory Society, pulmonary rehabilitation for a duration of 6–12 weeks is conditionally recommended for managing fatigue in patients with chronic sarcoidosis [84]. One observational pilot study evaluated the impact of a 12-week physical training program in 24 patients with IPF and fibrotic pulmonary sarcoidosis. Upon finishing the program, over 50% of patients had improvements in exercise capacity as assessed by 6-min walk distance, while others maintained their initial levels [110]. Another systematic review found that pulmonary rehabilitation may enhance exercise capacity and alleviate dyspnea in individuals with sarcoidosis, irrespective of stage of the disease [111]. These results highlight the potential in enhancing overall functional status among patients with sarcoidosis in general and with fibrotic pulmonary sarcoidosis. 

Patients with sarcoidosis have dysregulated immune responses caused by underlying granulomatous inflammation and concurrent use of immunosuppressive agents, which can affect the efficacy of vaccinations [112]. Considering this, the timing of immunosuppressive treatments must be taken into account when administering inactivated and live vaccines. In the case of live vaccinations, the benefits should be carefully weighed against the associated risks, and therapy should be temporarily delayed before and after administration of live vaccinations [112]. Especially with B-cell depleting therapies such as rituximab, vaccination dosing and frequency require careful consideration of scheduling [112,113]. 

Though the ERS treatment guidelines on sarcoidosis do not make specific recommendations regarding oxygen supplementation, patients with chronic hypoxemic respiratory failure due to pulmonary sarcoidosis should be supported with supplemental oxygen therapy [114]. Non-invasive ventilation may be used as supportive therapy in cases of respiratory failure. Currently, there is limited evidence regarding the potential benefits or risks associated with the use of supplemental oxygenation in pulmonary sarcoidosis, particularly concerning aspects such as nocturnal hypoxemia, exertional hypoxemia, dyspnea, and exercise endurance. Further studies in this area are essential for a comprehensive understanding of the implications and appropriate management strategies for these patients with chronic hypoxemic respiratory failure due to APS. 

### 5.8. Lung Transplantation

Lung transplantation serves as a final option for patients with fibrotic pulmonary sarcoidosis suffering from respiratory failure and pulmonary hypertension. According to International Society for Heart and Lung Transplantation (ISHLT) registry data, sarcoidosis accounts for 2.4% of all lung transplantations, and has a median survival of 6.1 years following transplantation [115,116]. There are no guidelines specifically tailored to sarcoidosis for lung transplantation; they currently follow those for ILD [117,118]. Following the implementation of the lung allocation score (LAS) in 2005, a greater percentage of sarcoidosis patients received lung allografts, leading to reduced waitlist mortality [119]. However, recent studies have found that, compared with patients with COPD and IPF, individuals with sarcoidosis continue to face disproportionately higher waitlist mortalities [120]. The several factors contributing to waitlist mortality were identified as pulmonary hypertension, poorer functional status, oxygen dependence, lower reduced output, and female sex [121,122]. Moreover, waitlisted patients’ percent predicted FVC was found to be significantly lower than the thresholds recommended by ISHLT lung transplant referrals, underscoring potential delays in referral for lung transplant [122]. Following transplantation, patients may experience increased perioperative morbidity and mortality attributed to higher rates of primary graft dysfunction, hemothorax, and prolonged dependence on ventilatory support [116,123,124,125]. Despite these initial risks, long-term survival rates appear to be comparable to those observed in other chronic lung conditions with a risk for disease recurrence [116,123,126]. Further research and development of more specific guidelines on selection and post-transplant management for patients with fibrotic pulmonary sarcoidosis are needed. 

## 6. Conclusions

Patients suffering from fibrotic pulmonary sarcoidosis experience higher morbidity and mortality compared with those without chronic and/or advanced disease. This may be due to the progressive nature of the disease with variable complications. Factors such as age, imaging findings, respiratory failure, and pulmonary hypertension may assist in prognostication, but this needs refinement and validation. The variability in disease presentation and progression makes determining the best approach for management challenging, and the approach should be individualized for each patient. There is a critical need to evaluate management strategies and continue research efforts aimed at improving patient outcomes and quality of life.
